# Regulation of hydrogen and oxidative stress in treating intestinal mucosa from ulcerative colitis

**DOI:** 10.1016/j.ijpx.2025.100429

**Published:** 2025-10-27

**Authors:** Na Yu, Jingwen Xu, Jie Fan, Huimin Gao, Ting Wu, Yunfeng Zhu, Jing Xu, Xiaolin Li, Huae Xu, Xiaowei Lu

**Affiliations:** a Department of Geriatrics, the First Affiliated Hospital of Nanjing Medical University, Nanjing, China; b Department of Pathology, Huadong Hospital, Fudan University, Shanghai, China; c Department of Geriatrics, the Jurong People's Hospital of Jiangsu University, Zhenjiang, China; d Department of Pharmaceutics, School of Pharmacy, Nanjing Medical University, Nanjing, China; e College of Materials Science and Engineering, Jiangsu Collaborative Innovation Centre for Advanced Inorganic Function Composites, Nanjing, China; f The Yancheng School of Clinical Medicine of Nanjing Medical University, Yancheng Third People's Hospital, Yancheng, China

**Keywords:** Ulcerative colitis, Magnesium hydride, Controlled release, Hydrogen, Oxidative stress, Inflammation

## Abstract

Ulcerative colitis (UC) is a diffuse chronic inflammation in the superficial intestine. Excessive accumulation of reactive oxygen species (ROS) leads to the of the colorectal damage. As the anti-inflammation therapy needs to be last for at least 3–5 years, the drugs demand less toxicity. However, current treatments in the clinic are often accompanied by unavoidable adverse side effects. Hydrogen is a nontoxic antioxidant reagent with excellent permeability of biomembranes, which shows the potential in treating UC. A novel simply designed hydrogen storage particle, Magnesium Hydride (MgH_2_) particle with an outer shell of passivated Magnesium oxide (MgO), was constructed in the current study to enable the safe and controlled release of hydrogen. This studies demonstrated that magnesium hydride particle (MgH_2_@MgO) was effective in scavenging excessive ROS, relieving the inflammation, and reversing the progression of UC through inhibiting the NF-*κ*B signaling pathway, which provided the experimental evidence for the clinical prevention and therapy of UC.

## Graphical abstract

1. The suspension of hydrogen storage particles was given by retention enema. The hydrolysis reaction releases hydrogen gas into the intestinal cavity slowly and continuously; 2. The gas passes through cell membrane via passive diffusion; 3. The hydrogen neutralizes reactive oxygen species (ROS) and reduces mucosal damage caused by neutralizing oxidative stress in ulcerative colitis.

**Figure:**
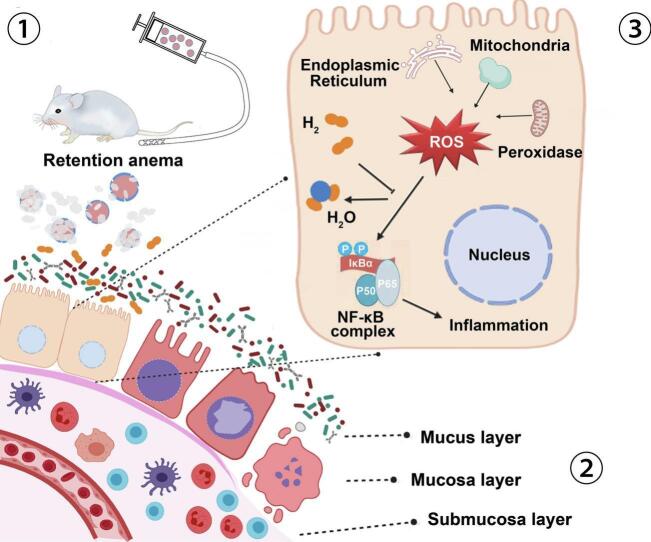

## 1 Introduction

Ulcerative Colitis (UC) is one of the inflammatory bowel diseases (IBD) which involves chronic inflammation from the rectum to ascending colon, characterized by constantly elevated reactive oxygen species (ROS). Multifactorial pathogenesis, including dysregulated immune responses, genetic predisposition, intestinal flora disorder, epithelial barrier damages, and environmental problems, could be account for the occurrence of UC. Relapsing diffuse mucosal inflammation and crypt abscesses in the colorectum are the characteristic pathological changes of UC (Le Berre et al., 2023). For patients diagnosed with UC for more than 8 years, the risk of developing colorectal cancer is 2–4 times higher than ordinary people. Recurrent mucosal lesions and repairment play an essential role in UC and the pathogenesis of UC-associated colorectal cancer (Segal et al., 2021). To effectively relieve colitis and prevent its recurrence patients need to take anti-inflammation treatments continuously for more than 3–5 years, even perpetually. However, taking medicine for a long time might cause liver function damage, in severe cases, it would damage the liver and seriously endanger the health of the body. Therefore, how to reduce the side effects of drugs is as essential as UC therapy in the long-term.

Hydrogen at a certain concentration is a reducing agent with no cytotoxicity and excellent permeability of biomembrane (Chuai et al., 2012). Usually, hydrogen, produced by intestinal colonization flora, plays an essential role in maintaining the balance of redox homeostasis in the gut. Compared to other antioxidants with the comparable antioxidant efficiency, the reductive activity of hydrogen was mild enough to maintain the physiological redox balance while producing negligible side effects to cells. As the electrically neutral gas, hydrogen could quickly dissolve in water, and readily penetrate membranes of cells and organelles, thereby neutralizing oxygen radicals efficiently. Many studies have proved that hydrogen treatment could effectively eliminate ROS accumulation, suppress inflammation, and inhibit tumor growth in many organs, such as the liver, brain, bone, and intestine (Le Berre et al., 2023). It is reported that this physiological gas could act as a substitute reducing agent, which only has therapeutic effects on cells with oxidative stress, such as inflammation or carcinoma, while showing a more negligible effect for normal tissue and cells (Dole et al., 1975). Zhu al et. reported that oral administration of acarbose alleviated the oxidative stress of ulcerative colitis by increasing intestinal flora-induced hydrogen production (Zhu et al., 2013). Another research found that intraperitoneal injection of hydrogen-rich saline could reduce experimental UC through decreasing vascular endothelial growth factor in rats (He et al., 2013). With the pleiotropic effects discussed above, hydrogen is considered to have good prospects for clinical application in IBD management (Le Berre et al., 2023).

A group of substances known as metal hydrides had the ability to store and release hydrogen under control. Because the end-product of Metal hydrides were pollution-free water, those compounds played an important role in green energy industries. Bio-reductive hydrogen derived from the hydrolysis of Metal and its hydride could realize controlled, durable, and slow release under physiological conditions (Zhang et al., 2019). Zhao et al. synthesized PdH_0.2_ nanocrystals as a hydrogen carrier to treat cancer through a synergetic hydrogen thermal therapy approach (Kawai et al., 2012). However, the Pd element had toxicity to the human body. Physiologically, magnesium (Mg), which was absorbed in the intestine, was not only an essential component of bone salt in the human body but also played an essential role in many physiological functions, such as ion channels regulation, smooth muscle excitability, and cell proliferation (Pelczyńska et al., 2022). Notably, IBD patients often suffered from hypomagnesemia, which could aggravate symptoms of colitis, increase genomic instability of intestinal mucosal cells and increase the risk of colorectal cancer (Pelczyńska et al., 2022). It has been reported that dietary Mg supplementation could safely and effectively alleviate inflammation, restore a beneficial intestinal flora, and suppress inflammation-associated carcinogenesis in the colon (Kuno et al., 2013).

Based on the versatile physiological effects and superior biocompatibility of magnesium ion, this study employed an excellent hydrogen generation material, magnesium hydride (MgH_2_), as a hydrogen generator (Zhao et al., 2012). The hydrolysis reaction between MgH_2_ and water could be described as follows:

(1) $${\mathrm{MgH}}_{2}+2H_{2}O\to \mathrm{Mg}{\left(\mathrm{OH}\right)}_{2}+2H_{2}$$

**Fig. 1:**
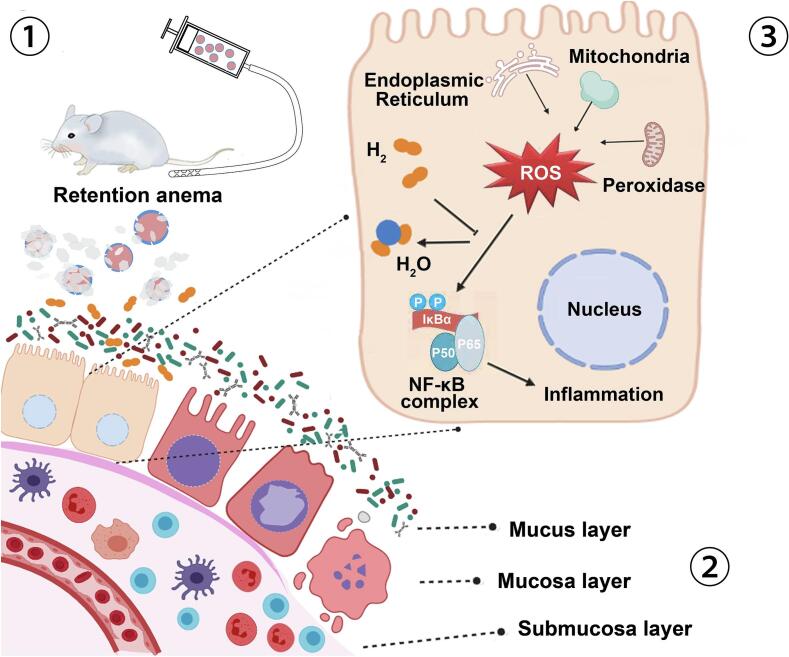
The scheme of MgH_2_@MgO in the treatment of ulcerative colitis. ①Give hydrogen storage particle suspension through retention enema. The hydrolysis reaction continuously and slowly releases hydrogen into the intestinal tract. ②Gases pass through the cell membrane in a passive diffusion manner. ③Hydrogen neutralizes reactive oxygen species (ROS), reducing mucosal damage caused by oxidative stress in ulcerative colitis.

Its theoretical hydrogen generation yield is 1703 mL g^−1^. Nevertheless, the releasing kinetics of hydrogen from free MgH_2_ was too fast to be controlled, which releasing kinetics of H_2_ was too fast to be controlled. Here we reported a novel simply designed hydrogen storage particle, MgH_2_ particles with an outer shell of passivated Magnesium oxide (MgO) to enable hydrogen's safe and controlled release (Fig. 1).

## 2 Materials and methods

### 2.1 Animals and cells

Male BABL/C mice (6 week old and 18–20 g) were provided by Nanjing Medical University Animal Center. All mice were raised under the SPF environments with a 12/12-h light/dark cycle at 21–23 °C according to the animal use guidelines given by National Institutes of Health. Animal experiments were conducted following the guidelines approved by the Ethics Committee of Nanjing Medical University (IACUC-2406018). Measures to minimize the suffering and the sample number of animals were conducted.

Human colorectal cancer cell line SW480 was cultured in sterile DMEM high glucose medium, which supplementing with 10 % FBS and 1 % penicillin-streptomycin solution. Cells were incubated in an atmosphere of humidified 5 % CO_2_ at 37 °C. The medium was changed every 2 d (except MTT assay).

### 2.2 Synthesis of MgH_2_@MgO

Original powder of Mg (99 wt% in purity, Tangshan Weihao Magnesium Powder Co.) was purchased commercially without any purification. The magnesium hydride particle (MgH_2_@MgO) was prepared by the hydriding combustion synthesis (HCS) method. The Mg powder was placed into the synthesis reactor, heated to 580 °C and held for 2 h (h). The reactor was naturally cooled down to 340 °C and held for 8 h. The hydrogen pressure in the synthesis reactor was kept at 2 MPa during the whole process. The reactor was frozen to room temperature and the sample of MgH_2_@MgO was obtained.

### 2.3 Hydrolysis test

The hydrolysis test was referred to our earlier research (Gan et al., 2018). The hydrolysis reaction was executed in a 50 mL flask with three openings: one for the hydrogen outlet, one for sealed with a plug and the last one for hydrolysis solution injection. The hydrogen generation by hydrolysis of MgH_2_@MgO in deionized water was determined by the drainage method at room temperature.

### 2.4 Characterization

X-ray diffraction with Cu Kα radiation (40 kV and 35 mA) was conducted to analyze the phase composition of the sample. Scanning electron microscope (SEM) was performed via JSM-6360LV to observe the morphology of samples.

### 2.5 Cell viability assay

The working suspensions were mixed by DMEM medium and MgH_2_MgO particles (0, 25, 50, 100, 200, 400 and 800 mg/L, respectively). To avoid the precipitation of particles and the loss of hydrogen, the working solution was used right after its ready. After 24, 48 and 72 h treatments, cells cultured in 96-well plates were incubated with 10 % 3-(4,5-dimethyl-2-thiazolyl)-2,5-diphenyl-2-H-tetrazolium bromide (MTT) for 4 h at 37 °C. Then the solutions were exchanged by dimethyl sulfoxide solution (100 μl/well) for 10 min (min). Absorbance at 570 nm was recorded using a microplate reader (Bio-Rad, USA). Cell viability was calculated as the average of three duplicate wells.

### 2.6 Clonogenic assay

Human colorectal cancer cell line SW480 were seeded in 6-well plate (200 cells/well) and cultured with MgH_2_@MgO at equivalent concentrations (0, 25, 50, 100 and 200 mg/L, respectively). The medium was gently changed every 2 d. After treatments for 7 d, the plates were fixed with 10 % formalin and then dyed with 0.5 % crystal violet for 10 min, respectively. Isolated cell clusters with 30–50 cells were counted as a colon.

### 2.7 Measurement of ROS production

The 2, 7-dichlorofluorescin diacetate (DCFH-DA) was used to analyze the intracellular oxidative stress. Lipopolysaccharide (LPS) was used for inducing inflammation in vitro. The SW480 cells were exposed to LPS in appropriate dose (20 mg/L) for 2 h before and following co-incubated with MgH_2_@MgO treatments. Cells were cultured with MgH_2_@MgO (0, 100 and 200 mg/L) for 4 h, respectively. Subsequently, each group was incubated with 10 μM DCFH-DA at 37 °C for 30 min. Fluorescent images of cells were taken under the laser confocal fluorescence microscopy. Intracellular green fluorescence was measured by flow cytometer.

### 2.8 JC-1 assay

The 5,5′,6,6′-tetrachloro-1,1′,3,3′-tetraethylbenzimidazolylcarbo cyanine iodide (JC-1) kit was used to measure the mitochondrial membrane potential (MMP). After 4 h of MgH_2_@MgO (0, 100 and 200 mg/L) treatments, the SW480 cells were incubated with JC-1 solution according to the instructions. The fluorescence intensity of JC-1 was measured using flow cytometer.

### 2.9 In vivo experiments

Male BABL/C mice were randomly divided into 3 groups (*n* = 7 mice/group), including the healthy control group (sodium carboxymethyl cellulose, CAC), the model group (dextran sulfate sodium, DSS) and the treating group (MgH_2_@MgO). For CAC group, mice drunk distilled water only. To induce acute ulcerative colitis, DSS group and MgH_2_@MgO group were fed with 2.5 % DSS solution as drinking water for 5 d, following with distilled water for 4 d (Chassaing et al., 2014). MgH_2_@MgO were suspended in 0.5 % sodium carboxymethyl cellulose (CAC) solution in a concentration of 200 mg/L and to use it right after it was ready. For MgH_2_@MgO group, the treatment was started at the 4th day. For healthy control group (CAC group) and DSS group, mice were given the same volume of 0.5 % CAC solution. Drugs were given to mice through retention enema (50 μl/mice) once a day. After enema, the mice keep inverted for 10 min to retain the drug and hydrogen. The treatment was last for 5 days. Disease activity index (DAI), as showed in Table 1, was used to estimate severity of colitis dynamically (Perera et al., 2018). Weight and survival rate of mice were recorded every day. On the 9th day, all mice were euthanized. The blood, fecal and colon tissues of mice were collected for further experiments. The fecal occult blood and the level of proteins were analyzed by ELISA kits according to the instruction.

**Table 1:** Assessment parameters of disease activity scores of experimental colitis.

Score	Weight loss (%)	Stool property	Fur coat	Hemoccult test
0	0	Normal	None	Negative
1	1–5	Loose stool	Rough coat	Positive
2	6–10	Diarrhea	–	Positive
3	11–15	Diarrhea and anal prolapse	–	Positive
4	>15	Diarrhea and blood	–	Positive

### 2.10 HE stain and immunohistochemistry

Colon tissues were fixed in neutral-buffered formalin (10 %) for 24 h, dehydrated through an ascending ethanol–xylene staircase and then embedded with paraffin. Sections were sliced in 4 μm, deparaffinised in two fresh xylene baths (7 min each) and rehydrated through graded ethanols. Nuclei were then stained with hematoxylin (6 min), differentiated in 0.3 % acid alcohol, and blued under warm tap water. Following a 30-s eosin-Y counterstain, slides were rapidly dehydrated, cleared in xylene, mounted with a synthetic resin and coverslipped. All sections were observed through a light microscope (Olympus, Japan) by two pathologists. The colorectal sections stained with hematoxylin and eosin were observed through optical microscope to evaluate those morphology changes. To promote antigen retrieval, the colorectal sections were autoclaved in citrate buffer for 2 min and then blocked in bovine serum albumin (5 %). Slices were incubated with primary antibodies against interleukin-6 (IL-6), tumor necrosis factor α (TNF-α), phosphorylation of protein P65 (pi-P65), 8-hydroxy-2′-deoxyguanosine (8-OHdG), myeloperoxidase (MPO) or GAPDH and further secondary antibodies according to the instructions. Subsequently, the tissues were visualized with diaminobenzidine, counterstained with hematoxylin solution. All sections were observed through a light microscope (Olympus, Japan) by two pathologists in a blinded manner. The acute colitis pathological criterion is shown in Table 2 (Kim et al., 2012).

**Table 2:** Assessment parameters of pathology score of colitis.

Score	Lesions
0	Normal
1	Focal inflammation
2	Multifocal inflammation or goblet cell depletion
3	Multifocal inflammation, damage of intestinal epithelial or severe crypt distortion
4	Multifocal inflammation, ulcer or loss of entire crypts

### 2.11 Statistical analysis

Outcomes were statistical expressed by Mean ± SD. Statistical significance was verified with one-way analysis of variance (ANOVA). The data on multiple time points were conducted using two-way repeated measure ANOVA. The analysis of non-categorical variables was conducted using Kruskal−Wallis test. *P* < 0.05 was considered as a significant difference. The corresponding analyses were conducted GraphPad prism9 software.

## 3 Results and discussion

### 3.1 Characterization of MgH_2_@MgO

**Fig. 2:**
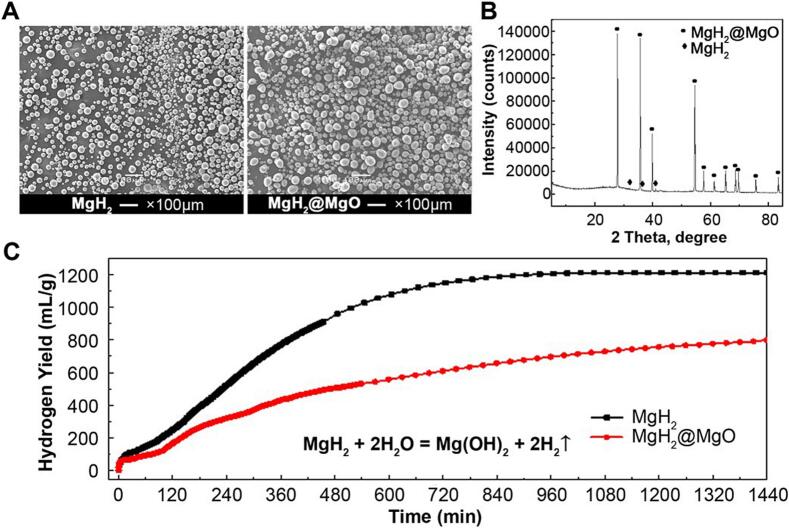
The characterization of MgH_2_@MgO (A) SEM images of MgH_2_ and MgH_2_@MgO. (B) Intensity of MgH_2_ and MgH_2_@MgO. (C) X-ray diffraction pattern of the sample prepared by hydriding combustion synthesis. (D) Hydrogen-release property of MgH_2_@MgO and free MgH_2_ in deionized water.

The analysis of magnesium oxide-coated magnesium hydride particle (*MgH*_*2*_*@MgO*) was shown in Fig. 2.

As shown in the SEM image, the shape of the original MgH_2_ powder was spherical, with a size of around 26.7 μm (Fig. 2A). After hydrogenation, the average size of MgH_2_@MgO increased to approximately 32.8 μm due to the volume expansion of the crystal lattice (Fig. 2B). The X-ray diffraction patterns of MgH_2_@MgO displayed in Fig. 2C indicated that the HCS product was mainly composed of MgH_2_@MgO with the trace of unhydrogenated Mg. According to the Rietveld analysis, the phase abundances of MgH_2_@MgO reached 99 wt%, showing the advantage of the HCS method to synthesize high purity MgH_2_. Magnesium hydride (MgH₂) is a typical metal hydride with high chemical reactivity. When in contact with water, it preferentially undergoes a non-enzymatic reaction to generate magnesium hydroxide and hydrogen gas. This reaction does not rely on enzymes and proceeds rapidly in aqueous systems (e.g., biological reaction environments). Upon dispersing into the aqueous phase, MgH_2_ particle could react vigorously with water and release a large amount of hydrogen, while the hydrolysis rate was significantly increased without the passivation of MgO (magnesium hydride coated with magnesium oxide). Around 763 mL hydrogen was obtained within 24 h for 1 g MgH_2_@MgO. The hydrolysis rate at 37 °C of the as-prepared MgH_2_@MgO in deionized water was relatively slow, and the hydrogen generation yield was approximately proportional to the reaction time. During 24 h of reaction, about 763 mL of hydrogen was generated by MgH_2_@MgO with an equivalent mass of 1 g MgH_2_. Effects of surface passivation realized the slow and stable release of continuous hydrogen.

### 3.2 Cytotoxicity of MgH_2_@MgO and LPS in vitro

**Fig. 3:**
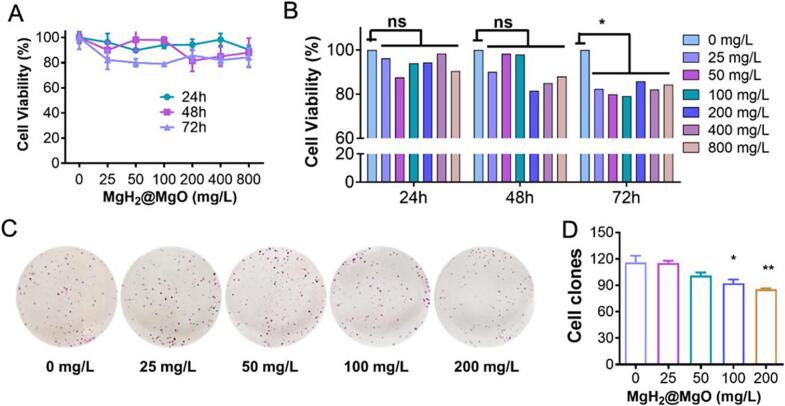
Cytotoxicity of MgH_2_@MgO particle (A) Cytotoxicity treated with MgH_2_@MgO. (B) Cellular activity assay of MgH_2_@MgO. (C) Plat clone formation assay of MgH_2_@MgO. (D) Cell viability value corresponding C. Data are mean ± SD; *P < 0.05, **P < 0.01 and ***P < 0.001 vs. Control. Intensity of MgH_2_ and MgH_2_@MgO. Each data were technical independently verified three times from 5 biological replicates for each strain.

As we know, intestinal flora disorder was one of the pathogenic factors in UC. LPS, as a standard component of the cytoderm of gram-negative bacteria, promoted the occurrence and development of UC (Candelli et al., 2021). As a strong exogenous stimulator, LPS could increase the level of ROS and induce cell damage. LPS was always used as an inducer of colitis for building biological models (Bao et al., 2023). In Fig. 3A-B, MgH_2_@MgO showed less cytotoxicity in the concentration from 0 mg/L to 800 mg/L.

Compared with the control group at the same time point, cellular proliferation was inhibited less than 20 % in all MgH_2_@MgO treated groups. The same trend of MgH_2_@MgO was also confirmed by the plate clone formation assay (Fig. 3C). The clonogenic ability of tumor cells was a sensitive indicator of drug cytotoxicity (Le Berre et al., 2023). In our test, cells exposed to the highest dose of 200 mg/L *MgH*_*2*_*@MgO* treatment showed a suppressed clone formation rate of 73.8 % relative to the control group (0 g/L MgH_2_) (Fig. 3D). Besides, LPS in low concentrations (0–5 mg/L) stimulated cell proliferation slightly while higher dosages of LPS led to a significant decrease in cell viability. Tomoaki Naito et al. reported that LPS played a dual role in colonic mucosa: on one hand, LPS suppressed cell proliferation by inducing necroptosis, on the other hand, it promoted differentiation of the goblet cell lineage (Naito et al., 2017)**.**

In this study, the abnormal rise was probably associated with the MgH_2_@MgO-suppressed oxidative stress, which protected cells from LPS-induced cytotoxicity. These experiments confirmed the low cytotoxicity of MgH_2_@MgO in colon cells and demonstrated the protective ability of MgH_2_@MgO from inflammatory damage in a dose-dependent manner.

### 3.3 Efficient suppression of oxidative stress in cells treated with MgH_2_@MgO

**Fig. 4:**
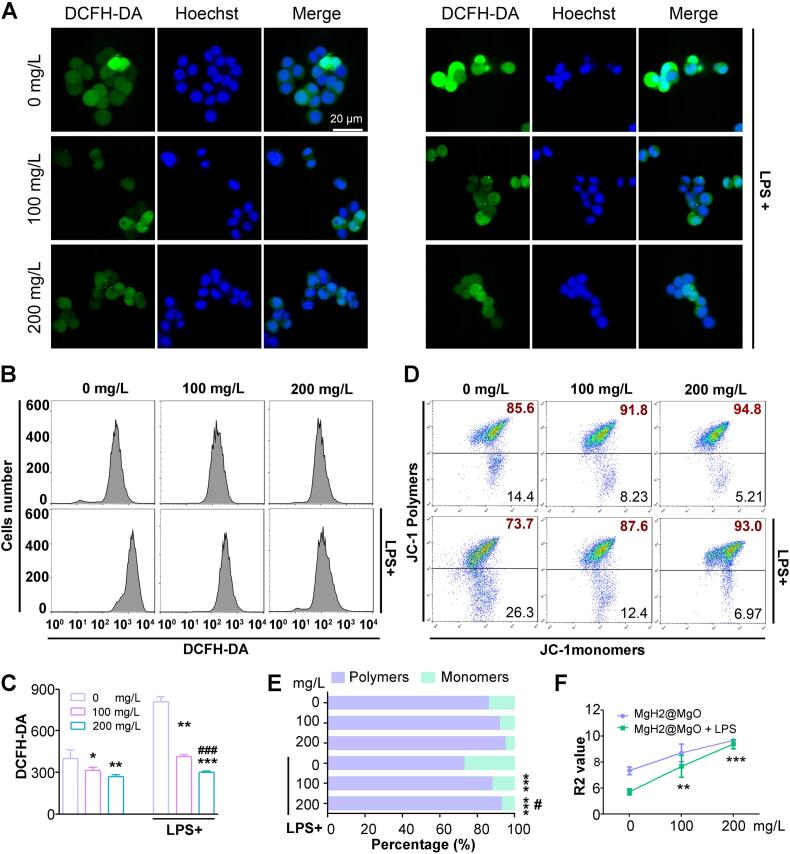
MgH_2_@MgO treatment decreased the intracellular ROS level and protected cells from mitochondrial dysfunction (A) Evaluation of intracellular ROS level by the laser confocal fluorescence microscopy. (B) Intracellular ROS levels after treatment by MgH_2_@MgO with or without LPS. (C) Fluorescence intensity of DCFH. (D) Flow cytometry analysis of levels of JC-1 polymers and monomers. (E) Ratios of fluorescence intensities indicating JC-1 polymers/monomers (R2 Value). (F) Fluorescence intensity of polymers/monomers of JC-1 in cells after different treatments Data are mean ± SD; *P < 0.05, **P < 0.01 and ***P < 0.001 vs. control group. # *P < 0.05* and ### *P <* 0.001 vs. model group. Scale bar = 20 μm. Each data were technical independently verified three times from 5 biological replicates for each strain.

The oxidative stress is one of the dominant pathological mechanism that induces cell damage in UC (Blagov et al., 2023)1. ROS, causing by epithelial barrier damage and neutrophil immune dysfunction, are the direct conditioners and activators in inflammation tissue. Anti-oxidant therapy seems to be a promising directions (Aviello and Knaus, 2018). MgH₂ is a strongly reducing substance, while ROS, including superoxide anion (O₂•^−^), hydrogen peroxide (H₂O₂) and hydroxyl radical (•OH), are typical amphoteric substances in redox reactions. Rather than a simple hydrolysis reaction，when the two substances come into contact, ROS will preferentially act as an oxidizing agent, and MgH₂ will act as a reducing agent, undergoing a redox reaction with clear electron transfer. 2,7-dichlorodi-hydrofluorescein diacetate (DCFH-DA) probe was commonly used as an indicator of intracellular ROS generation (Eruslanov and Kusmartsev, 2010)23. In Fig. 4A-B, the highest fluorescence intensity showed LPS significantly elevated ROS levels of SW480 cells, which was inhibited after MgH_2_@MgO incubation. Quantitative flow cytometric analysis **(**Fig. 4C) demonstrated that MgH_2_@MgO inhibited the inherently high ROS level of cancer cells (*P* < 0.05). For higher ROS levels induced by LPS, MgH_2_@MgO significantly reduced it to a lower concentration than the typical levels of tumor cells in a dose-depended manner (*P* < 0.05). The results suggested that MgH_2_@MgO possibly provided a protective effect on cells from inflammation through suppressing intracellular oxidative stress.

Mitochondrial dysfunction was a reaction to intracellular oxidative damage in the early stage, which sensitively resulted from increased oxidative stress. One of its earliest phenomena was the decline of electrochemical gradient across the MMP TheJC-1 probe was a sensitive indicator to evaluate the MMP. In normal cells, JC-1 existed as polymers in the mitochondrial matrix which emitted red fluorescence, indicating a high level of MMP (Xu et al., 2025). In the presence of oxidative stress, a decrease of the MMP induced the depolymerization of JC-1 from polymers to monomers, producing green fluorescence. The red/green fluorescence (R2 value) indicated mitochondrial dysfunction. In this study, the MMP value was decreased in LPS exposed group, while the addition of MgH_2_@MgO treatments could up-regulate the MMP and reverse the oxidative damage (Fig. 4D-F) (*P <* 0.01).

### 3.4 MgH_2_@MgO reduced oxidative stress via NF-κB pathway

**Fig. 5:**
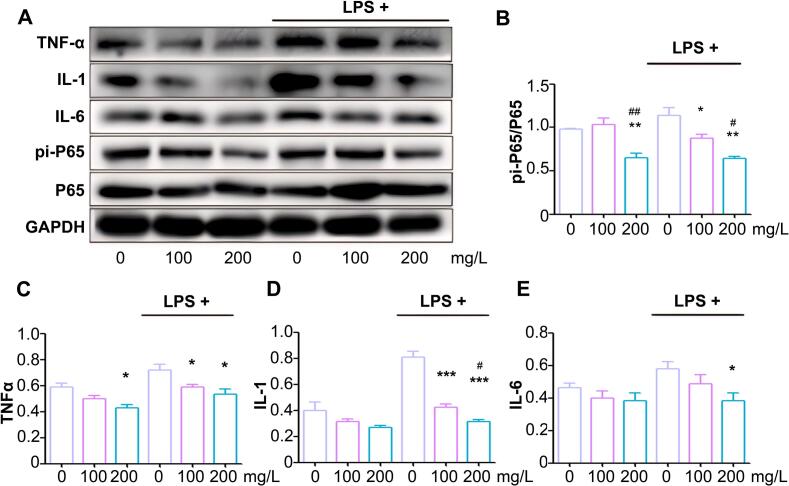
MgH_2_@MgO reduced protein expression of NF-κB pathway (A) Expression of proteins in colorectal tissue. (B) Quantitative ratio of phosphorylated P65 protein to total P65 protein. (C, D and E) Protein expression of TNF-α (C), IL-1 (D) and IL-6 (E) to GAPDH ratio. Data are mean ± SD; *P < 0.05, **P < 0.01 and ***P < 0.001 vs. control group. # *P < 0.05* and ## *P <* 0.01 vs. model group. Each data were technical independently verified three times from 5 biological replicates for each strain.

At a certain low concentration, LPS stimulated cell growth in a time-dependent manner, and many kinds of research confirmed that this process was mediated through activating the transcription factor nuclear factor kappa B (NF-κB) pathway (Mao et al., 2025). The NF-κB pathway was an important signaling pathway in regulating inflammation, which was involved in maintaining the intestinal immune system homeostasis and epithelial barrier 27^41^. The signaling cascade of the NF-κB pathway was a central modulator of UC pathogenesis (Bahrami et al., 2024). In the intestine, persistently high levels of ROS abnormally activated the (RelA/p65)/p50 heterodimers of NF-κB, further promoted NF-κB nuclear translocation and the following downstream genes expression, such as TNF-α, IL-1and IL-6 (McDaniel et al., 2016). The high level of NF-κB proteins expression was found in the intestinal tissues of UC patients (Makaro et al., 2020). Reducing the level of intracellular oxidative stress or blocking the NF-κB pathway effectively inhibited inflammation in vivo and in vitro (Zhao et al., 2024) Proteins of NF-κB pathway and inflammatory factors (IL-1, IL-6, and TNF-α) were detected to explore the molecular mechanism of MgH_2_@MgO therapy in colitis (Fig. 5A). In Fig. 5C-E, MgH_2_@MgO treatment down-regulated inflammatory factors. Meanwhile, MgH_2_@MgO inhibited the activation of NF-κB signaling pathway (Fig. 5B). Compared with the control group, the phosphorylate ratio of P65 (pi-P65/P65) was up to 116.6 % in the LPS group, indicating activation of the NF-κB pathway in colitis. P65 phosphorylation was inhibited after MgH_2_@MgO treatments, with a ratio of 75.9 % in the 200 mg/L MgH_2_@MgO group and 88.6 % in the LPS + 200 mg/L MgH_2_@MgO group which showed a dose-dependent expression of the inflammatory factor.

### 3.5 MgH_2_@MgO treatment protected mice from DSS-induced UC

**Fig. 6:**
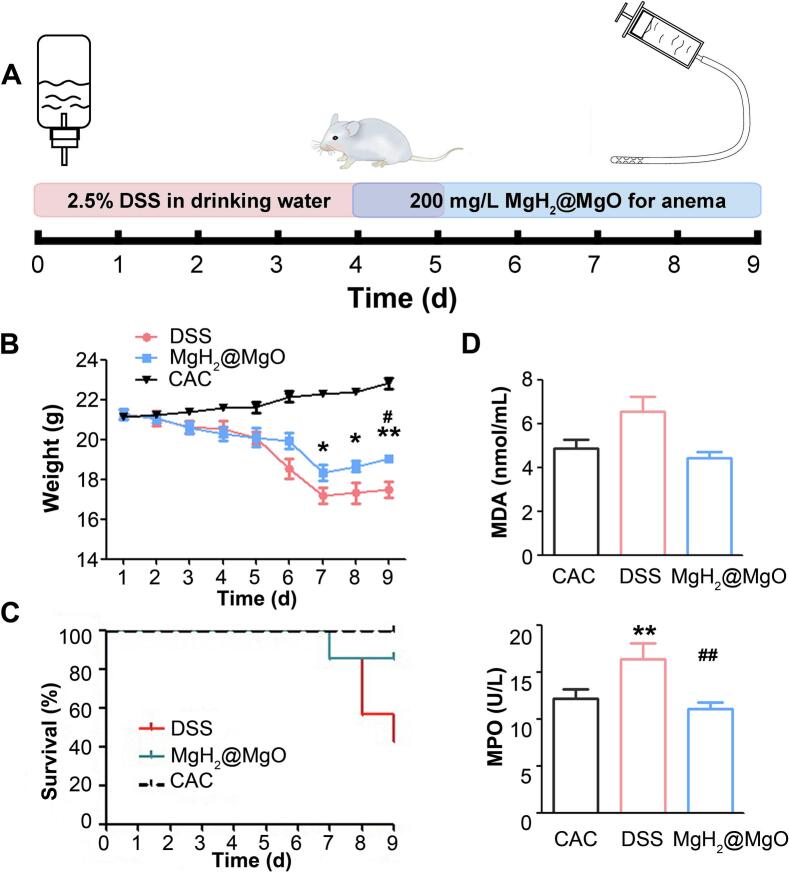
MgH_2_@MgO treatment protected mice from DSS-induced UC (A) Treatment illustration on mic. (B) Dynamic and terminal bodyweight of mice. (C) Dynamic survival rate of mice. (D) Oxidative stress-related biomarkers in blood. Data are mean ± SD, *n* = 7; **P < 0.01 vs. control group. # *P < 0.05* and ## *P <* 0.01 vs. model group.

To further explore the therapeutic effect of MgH_2_@MgO, mice models of acute ulcerative colitis were treated with MgH_2_@MgO (200 mg/L) suspension by daily retention enema (Fig. 6A).

Retention enema was an effective treatment for patients with mild to moderate UC, with immediate and direct therapeutic effect on mucous and without systemic side effects (Gordon et al., 2023). However, oral Mg salts were reported as a volumetric laxative (Di Palma et al., 2021). In this study, the final compound produced was magnesium hydroxide with low water solubility, which was difficult to form free Mg ions with effective concentration. Considering the inaccessibility of oral MgH_2_@MgO treatment to arrive at the colorectum with enough therapeutic concentration, the suspensions were given through retention enema therapy. The hydrolysate, hydrogen gas, could be discharged from the natural body cavity in time. Notably, comparing with traditional enema drugs, MgH_2_@MgO had a wider therapeutic area due to the free diffusion of hydrogen gas. The survival rate, body weight, and disease severity of mice were observed during this experiment. At the terminal of the experiment, inflammatory markers in blood and colon tissue were analyzed by ELISA test. Compared with the colitis (DSS) group, mice treated with MgH_2_@MgO (MgH_2_@MgO group) had slower weight loss and less mortality (Fig. 6B-C) (*P* < 0.05). The body weight rose in physiological rate in the healthy control group, which was given an enema of CAC solution, while obviously decreasing after DSS feeding. For the last 3 days of the experiment, the weight of mice was slightly raised up in both the DSS group and the MgH_2_@MgO group, possibly due to the self-healing ability after the DSS withdrawal and the death of severely-ill mice.

Biomarkers of inflammation and oxidative stress were extracted from the serum. Malondialdehyde (MDA), one of the end products of lipid peroxidation, is widely used to reflect the degree of imbalance in the ROS system in vivo ^47^. Myeloperoxidase (MPO), a heme enzyme rich in myeloid cells, plays an important role in anti-infection process and is also a common indicator of oxidative stress and inflammatory damage. The levels of MPO(*P* < 0.05) and MDA were significantly raised in DSS-treated mice and could be inhibited by MgH_2_@MgO treatment (Fig. 6D). For more systematic assessment of colitis, length of total colon and DAI of the colon were commonly used to evaluate the severity of colitis. DAI was analyzed every day according to Table 1.

**Fig. 7:**
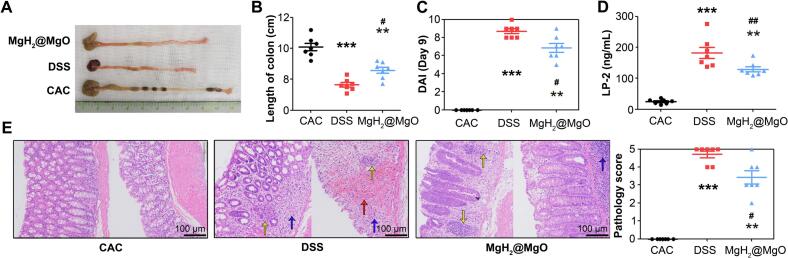
Pathological changes in MgH_2_@MgO treatment colitis (A) Image of the colon length. (B) Length analysis colons in the treating group (MgH_2_@MgO), model group (DSS), and healthy control group (CAC). (C) Disease activity index in the groups of MgH_2_@MgO, DSS, and CAC. (D) LP-2 protein expression in colon. (E) Images and pathology score in colorectal tissue. Data are mean ± SD, *n* = 7; *P < 0.05 and **P < 0.01 vs. control group. # *P < 0.05* and ## *P <* 0.01 vs. model group. Scale bar = 100 μm.

We also analyzed pathological changes and the expression of lipocalin-2 (LP-2) in colon tissue. The length of the colon is related to the degree of intestinal inflammation. In Fig. 7A-B, the DSS group showed the shortest colon length, indicating the most serious colitis (*P <* 0.05). In contrast, MgH_2_@MgO treatment could protect mice from acute colitis. The DAI of the CAC group was obviously and continuously increased after colitis induction (Fig. 7C). Regularly MgH_2_@MgO retention enema could partly relieve colitis in mice (*P <* 0.05). (LP-2, a neutrophil gelatinase associated lipocalin, amplifies inflammation and causes tissue damage by activating the NF-κB/NLRP3 pathway. LP-2 is markedly increased in inflammatory colonic epithelial tissue from UC patients (Yang et al., 2024). The expression of colitis associated protein LP-2 (Fig. 7D) proved the remission of inflammation in the MgH_2_@MgO group (*P <* 0.01). The colorectal tissues were stained with hematoxylin and eosin and independently estimated by two pathological doctors according to the pathological score of colitis (Table 2). The characteristic pathomorphological change (Fig. 7E) of UC included destruction of intestinal epithelial gland structure, infiltration of the inflammatory cell (blue arrow), hemorrhage (red arrow) and even crypt abscess (yellow arrow). Diffuse and severe histological damage could be observed in the DSS group. The destruction of colorectal epithelial structure lead to a serious intestinal barrier dysfunction. In contrast, only slightly gland destruction and inflammatory infiltration were found in the MgH_2_@MgO group, indicating that MgH_2_@MgO can improve intestinal barrier function (*P <* 0.05).

**Fig. 8:**
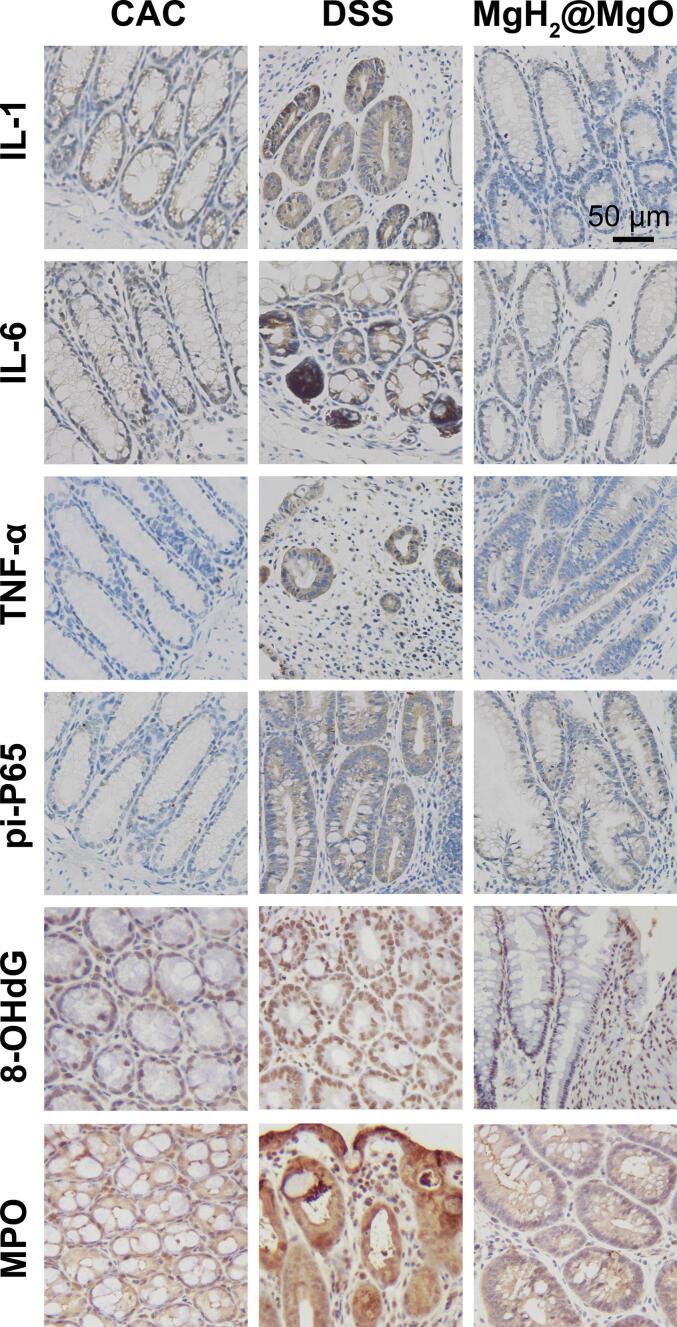
Protein expression in colorectal tissue. Scale bar = 50 μm.

It was reported that overproduction of ROS is a key pathogenic factor of UC. Overload of oxygen radical attributes to cellular dysfunction and intestinal barrier destruction (Aviello and Knaus, 2017). The NF-κB pathway was one of the main pathways to modulate colorectal inflammation and oxidative stress. Both accumulation of ROS and activation of the NF-κB pathway are detected in UC. Suppressing ROS is an efficient anti-inflammatory therapy (Blagov et al., 2023). Even though there were many anti-oxidative drugs available, the instability and side effects of reducing agents still restricted their potential application in clinical (Zhang et al., 2022) (Larabi et al., 2020). In Fig. 8, the immunohistochemistry images showed elevated levels of inflammatory factors in the DSS group. These protein expressions were highly inhibited in MgH_2_@MgO treated colitis, explicated an effective suppression of colorectal inflammation. It was reported that oxidative stress induced permanent damage in cellular membranes, proteins, and DNA lipids. 8-OHdG has been widely used to assess the oxidative damage induced by free radicals (Muro et al., 2024). MPO was a general peroxidase mainly produced by neutrophils. Local expression of MPO could induce catalyzes the production of potent ROS. MPO protein was also increased in mucosa of UC as an indicator of oxidative damage (Atay and Canbakan, 2025). Many MPO inhibitors have been studied as a targeted therapy of several chronic inflammations (Tangeten et al., 2022). In this study, MgH_2_@MgO group showed the decreased expression of both 8-OHdG and MPO stimulated by DSS-colitis. Meanwhile, the excessive pi-P65 in the DSS group and its inhibition in MgH_2_@MgO group supported that MgH_2_@MgO therapy played the role in the antioxidant and anti-inflammatory effect partly via the NF-κB pathway.

In current study, the SW480 cell line and mice were used to explore the UC. While these models have provided valuable insights into acute inflammation of colon, may not fully recapitulate the complexity of in vivo chronic inflammation. Another critical limitation in our study relates to the drug retention time. As we know, the intestine has continuous physiological peristalsis. In the mice model, we delayed drug elimination by hanging upside down for 10 min after administration. Despite these limitations, our study constructed a novel antioxidant compound named MgH_2_@MgO, with stable hydrogen storage and controllable release capacities. MgH_2_@MgO could slowly release hydrogen in water and showed a long-lasting antioxidant effect both in vitro and in vivo. We observed an indispensable anti-inflammation effect of MgH_2_@MgO in LPS-induced inflammatory colon cells. In mice with US, MgH_2_@MgO retention enema obviously reduced the free radical damage to colorectum, suppressed the development of colitis, relieved the localized pathological change and the systemic reaction, even decreased the fatality rate. Experimental models of UC demonstrated that the abnormal activation of the NF-κB pathway could be inhibited by MgH_2_@MgO treatment. Besides, the biological characteristic analysis of MgH_2_ showed its minimal cytotoxicity in a rational dose range. As a biocompatible antioxidant agent with controllable hydrogen release property, MgH_2_@MgO showed its clinical potential for long-term and stable therapeutical effect on UC.

## 4 Conclusions

In summary, this study constructed a novel antioxidant compound named MgH_2_@MgO, with stable hydrogen storage and controllable release capacities. MgH_2_@MgO could slowly release hydrogen in water and showed a long-lasting antioxidant effect both in vitro and in vivo. We observed an indispensable anti-inflammation effect of MgH_2_@MgO in LPS-induced inflammatory colon cells. In mice with US, MgH_2_@MgO retention enema obviously reduced the free radical damage to colorectum, suppressed the development of colitis, relieved the localized pathological change and the systemic reaction, even decreased the fatality rate. Experimental models of UC demonstrated that the abnormal activation of the NF-κB pathway could be inhibited by MgH_2_@MgO treatment. Besides, the biological characteristic analysis of MgH_2_ showed its minimal cytotoxicity in a rational dose range. As a biocompatible antioxidant agent with controllable hydrogen release property, MgH_2_@MgO showed its clinical potential for long-term and stable therapeutical effect on UC.

## CRediT authorship contribution statement

**Na Yu:** Writing – original draft, Investigation, Conceptualization. **Jingwen Xu:** Methodology, Investigation. **Jie Fan:** Methodology. **Huimin Gao:** Methodology. **Ting Wu:** Investigation. **Yunfeng Zhu:** Writing – original draft, Visualization. **Jing Xu:** Conceptualization. **Xiaolin Li:** Writing – review & editing. **Huae Xu:** Writing – review & editing, Funding acquisition, Conceptualization. **Xiaowei Lu:** Writing – review & editing, Supervision, Resources, Funding acquisition, Conceptualization.

## Declaration of competing interest

The authors declare no conflicts of interest.

## Data Availability

Data will be made available on request.

## Appendix A Supplementary data

Supplementary material Image 1

## References

[bb0005] Atay K., Canbakan B. (2025). Assessment of oxidative stress parameters for evaluation of disease activity in patients with ulcerative colitis. J. Physiol. Pharmacol..

[bb0010] Aviello G., Knaus U.G. (2017). ROS in gastrointestinal inflammation: rescue or Sabotage?. Br. J. Pharmacol..

[bb0015] Aviello G., Knaus U.G. (2018). NADPH oxidases and ROS signaling in the gastrointestinal tract. Mucosal Immunol..

[bb0020] Bahrami A., Khalaji A., Bahri Najafi M., Sadati S., Raisi A., Abolhassani A., Eshraghi R., Khaksary Mahabady M., Rahimian N., Mirzaei H. (2024). NF-κB pathway and angiogenesis: insights into colorectal cancer development and therapeutic targets. Eur. J. Med. Res..

[bb0025] Bao P., Gong Y., Wang Y., Xu M., Qian Z., Ni X., Lu J. (2023). Hydrogen sulfide prevents LPS-induced depression-like behavior through the suppression of NLRP3 inflammasome and pyroptosis and the improvement of mitochondrial function in the Hippocampus of mice. Biology.

[bb0030] Blagov A.V., Orekhova V.A., Sukhorukov V.N., Melnichenko A.A., Orekhov A.N. (2023). Potential use of antioxidant compounds for the treatment of inflammatory bowel disease. Pharmaceuticals (Basel, Switzerland).

[bb0035] Candelli M., Franza L., Pignataro G., Ojetti V., Covino M., Piccioni A., Gasbarrini A., Franceschi F. (2021). Interaction between lipopolysaccharide and gut microbiota in inflammatory bowel diseases. Int. J. Mol. Sci..

[bb0040] Chassaing B., Aitken J.D., Malleshappa M., Vijay-Kumar M. (2014). Dextran sulfate sodium (DSS)-induced colitis in mice. Curr. Protoc. Immunol..

[bb0045] Chuai Y., Qian L., Sun X., Cai J. (2012). Molecular hydrogen and radiation protection. Free Radic. Res..

[bb0050] Di Palma J.A., Bhandari R., Cleveland M.V., Mishkin D.S., Tesoriero J., Hall S., McGowan J. (2021). A safety and efficacy comparison of a new sulfate-based tablet bowel preparation versus a PEG and ascorbate comparator in adult subjects undergoing colonoscopy. Am. J. Gastroenterol..

[bb0055] Dole M., Wilson F.R., Fife W.P. (1975). Hyperbaric hydrogen therapy: a possible treatment for cancer. Science (New York, N.Y.).

[bb0060] Eruslanov E., Kusmartsev S. (2010). Identification of ROS using oxidized DCFDA and flow-cytometry. Methods Mol. Biol..

[bb0065] Gan D., Liu Y., Zhang J., Zhang Y., Cao C., Zhu Y., Li L. (2018). Kinetic performance of hydrogen generation enhanced by AlCl3 via hydrolysis of MgH2 prepared by hydriding combustion synthesis. Int. J. Hydrog. Energy.

[bb0070] Gordon H., Biancone L., Fiorino G., Katsanos K.H., Kopylov U., Al Sulais E., Axelrad J.E., Balendran K., Burisch J., de Ridder L. (2023). ECCO guidelines on inflammatory bowel disease and malignancies. J. Crohns Colitis.

[bb0075] He J., Xiong S., Zhang J., Wang J., Sun A., Mei X., Sun X., Zhang C., Wang Q. (2013). Protective effects of hydrogen-rich saline on ulcerative colitis rat model. J. Surg. Res..

[bb0080] Kawai D., Takaki A., Nakatsuka A., Wada J., Tamaki N., Yasunaka T., Koike K., Tsuzaki R., Matsumoto K., Miyake Y. (2012). Hydrogen-rich water prevents progression of nonalcoholic steatohepatitis and accompanying hepatocarcinogenesis in mice. Hepatology.

[bb0085] Kim J.J., Shajib M.S., Manocha M.M., Khan W.I. (2012). Investigating intestinal inflammation in DSS-induced model of IBD. J. Visual. Exp..

[bb0090] Kuno T., Hatano Y., Tomita H., Hara A., Hirose Y., Hirata A., Mori H., Terasaki M., Masuda S., Tanaka T. (2013). Organomagnesium suppresses inflammation-associated colon carcinogenesis in male Crj: CD-1 mice. Carcinogenesis.

[bb0095] Larabi A., Barnich N., Nguyen H.T.T. (2020). New insights into the interplay between autophagy, gut microbiota and inflammatory responses in IBD. Autophagy.

[bb0100] Le Berre C., Honap S., Peyrin-Biroulet L. (2023). Ulcerative colitis. Lancet.

[bb0105] Makaro A., Fichna J., Włodarczyk M. (2020). Single nucleotide polymorphisms in colitis-associated colorectal cancer: a current overview with emphasis on the role of the associated genes products. Curr. Drug Targets.

[bb0110] Mao H., Zhao X., Sun S.C. (2025). NF-κB in inflammation and cancer. Cell. Mol. Immunol..

[bb0115] McDaniel D.K., Eden K., Ringel V.M., Allen I.C. (2016). Emerging roles for noncanonical NF-κB signaling in the modulation of inflammatory bowel disease pathobiology. Inflamm. Bowel Dis..

[bb0120] Muro P., Zhang L., Li S., Zhao Z., Jin T., Mao F., Mao Z. (2024). The emerging role of oxidative stress in inflammatory bowel disease. Front. Endocrinol..

[bb0125] Naito T., Mulet C., De Castro C., Molinaro A., Saffarian A., Nigro G., Bérard M., Clerc M., Pedersen A.B., Sansonetti P.J. (2017). Lipopolysaccharide from crypt-specific core microbiota modulates the colonic epithelial proliferation-to-differentiation balance. mBio.

[bb0130] Pelczyńska M., Moszak M., Bogdański P. (2022). The role of magnesium in the pathogenesis of metabolic disorders. Nutrients.

[bb0135] Perera A.P., Fernando R., Shinde T., Gundamaraju R., Southam B., Sohal S.S., Robertson A.A.B., Schroder K., Kunde D., Eri R. (2018). MCC950, a specific small molecule inhibitor of NLRP3 inflammasome attenuates colonic inflammation in spontaneous colitis mice. Sci. Rep..

[bb0140] Segal J.P., LeBlanc J.F., Hart A.L. (2021). Ulcerative colitis: an update. Clin. Med. (Lond.).

[bb0145] Tangeten C., Zouaoui Boudjeltia K., Delporte C., Van Antwerpen P., Korpak K. (2022). Unexpected role of MPO-oxidized LDLs in atherosclerosis: in between inflammation and its resolution. Antioxidants (Basel, Switzerland).

[bb0150] Xu S., Wu L., Chen B., Deng X., Zheng Z., Wu F., Zeng L., Zheng C., Hu X., Huang A. (2025). A self-delivery albumin nanomedicine amplified photodynamic therapy against esophageal cancer through COX-2/PGE2 interruption and regulation of mitochondrial respiratory. Int. J. Pharm. X.

[bb0155] Yang Y., Li S., Liu K., Zhang Y., Zhu F., Ben T., Chen Z., Zhi F. (2024). Lipocalin-2-mediated intestinal epithelial cells pyroptosis via NF-κB/NLRP3/GSDMD signaling axis adversely affects inflammation in colitis. Biochim. Biophys. Acta.

[bb0160] Zhang L., Zhao P., Yue C., Jin Z., Liu Q., Du X., He Q. (2019). Sustained release of bioactive hydrogen by Pd hydride nanoparticles overcomes Alzheimer's disease. Biomaterials.

[bb0165] Zhang C., Wang H., Yang X., Fu Z., Ji X., Shi Y., Zhong J., Hu W., Ye Y., Wang Z. (2022). Oral zero-valent-molybdenum nanodots for inflammatory bowel disease therapy. Sci. Adv..

[bb0170] Zhao Z., Zhu Y., Li L. (2012). Efficient catalysis by MgCl2 in hydrogen generation via hydrolysis of Mg-based hydride prepared by hydriding combustion synthesis. Chem. Commun. (Camb.).

[bb0175] Zhao Z., Yi S., E H., Jiang L., Zhou C., Zhao X., Yang L. (2024). α-amanitin induce inflammatory response by activating ROS/NF-κB-NLRP3 signaling pathway in human hepatoma HepG2 cells. Chemosphere.

[bb0180] Zhu J.H., Zhang D.Q., Chen W.C. (2013). Managing ulcerative colitis by increasing hydrogen production via oral administration of Acarbose. Afr. J. Tradit. Complement. Altern. Med..

