# Supplementary material for: Regulation of hydrogen and oxidative stress in treating intestinal mucosa from ulcerative colitis
Source: Int J Pharm X. 2025 Oct 27;10:100429. doi: 10.1016/j.ijpx.2025.100429 (PMC12617788; doi:10.1016/j.ijpx.2025.100429)

**Supporting Information**.

***Supplementary Methods***

Hemolysis assay

Fresh human RBCs (2 % v/v) were incubated 37 °C 1 h with 100 mg/L MgH_2_, 200 mg/L MgH_2_, 0 % NaCl (negative control), or 1 % Triton X-100 (positive control).

**Supplementary** **figuress**

***Supplementary figure 1.*** The in *vitro* cytotoxicity of free MgH_2_

The cell viability (%) of free MgH_2_ shown obvious cytotoxicity in SW480 cells for 24 h. Each point presented as mean ± SD (n = 5).


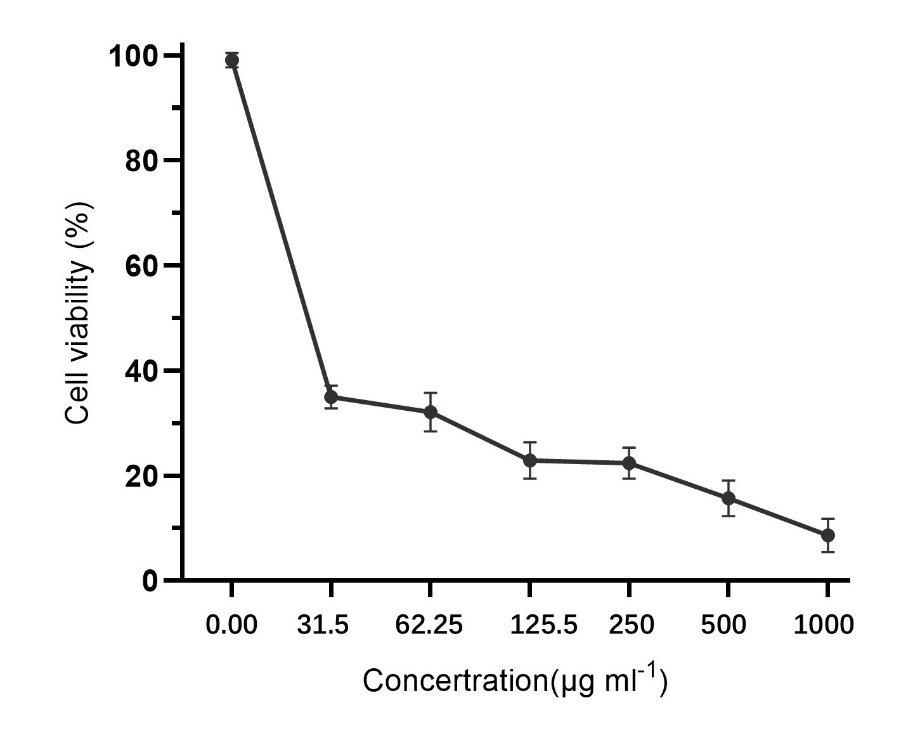


***Supplementary figure 2.*** The hemolysis assay of free MgH_2_

The hemolysis assay shown positive for 100 mg/L and 200 mg/L free MgH_2_.


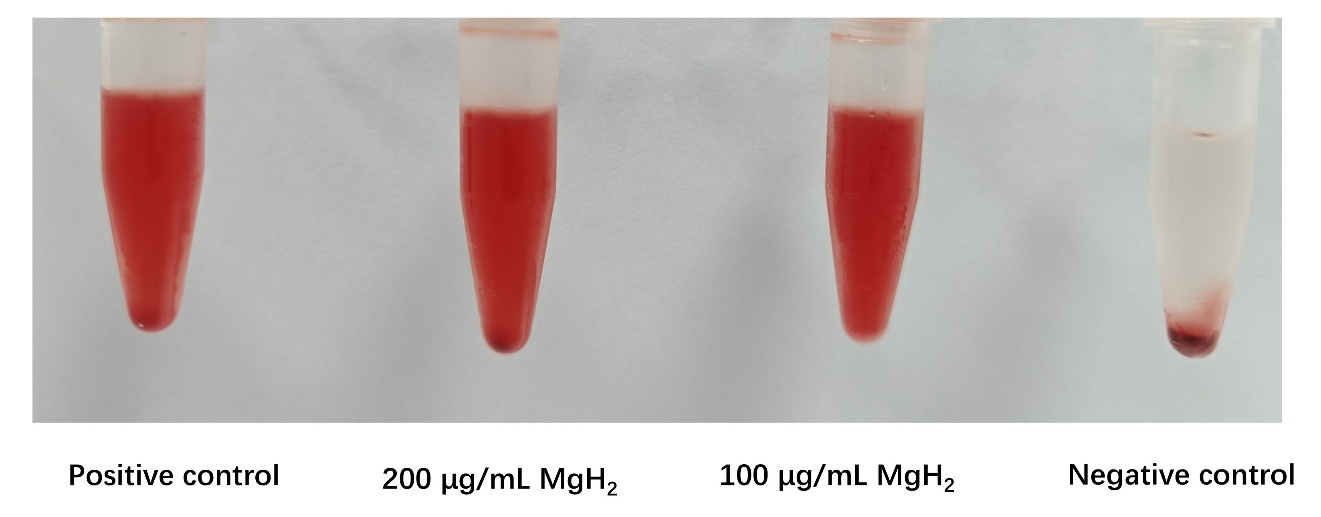


**Supplementary figure 3.** The dynamic survival rate of the DSS-induced colitis in mice

A 5-day UC models with different DSS concentrations (1%, 2%, 2.5%, 5%) exhibited different mortality rates with a treatment of 0.9% sodium chloride solution enema (n=3).


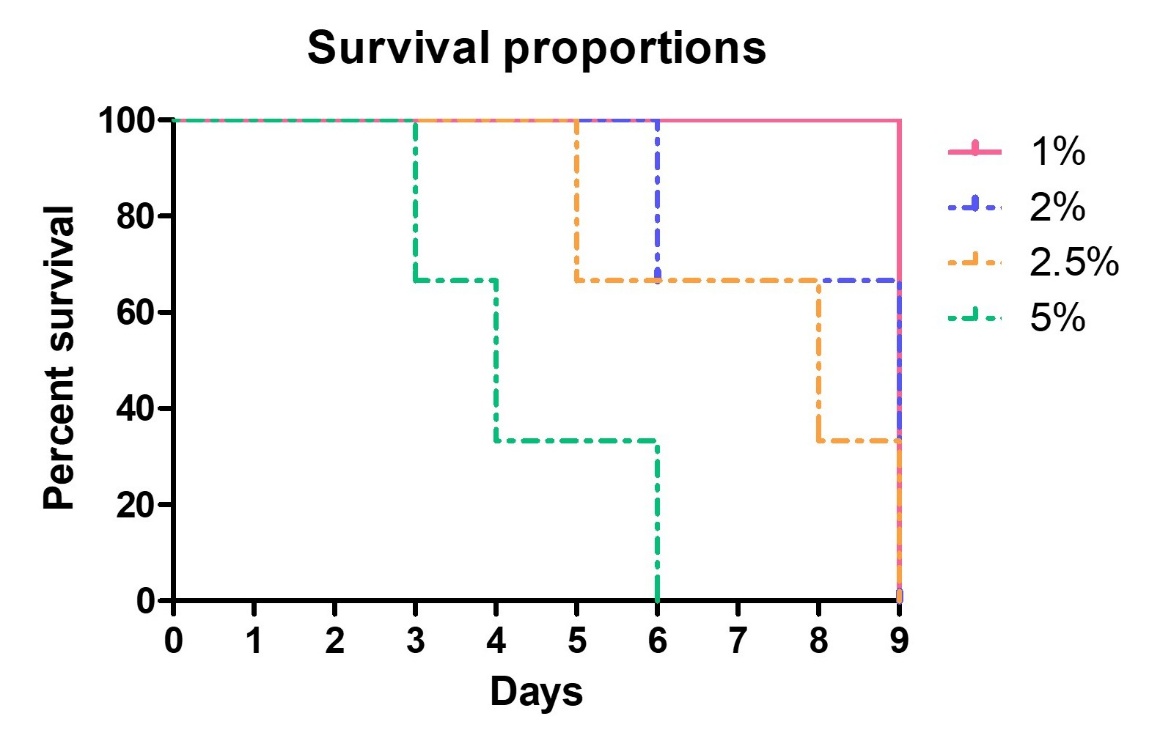


**Supplementary figure 4.** Disease activity index in the DSS-induced colitis in mice

The 5-day UC models with different DSS concentrations (1%, 2%, 2.5%, 5%) shown varying active colorectal inflammation in Day 5.


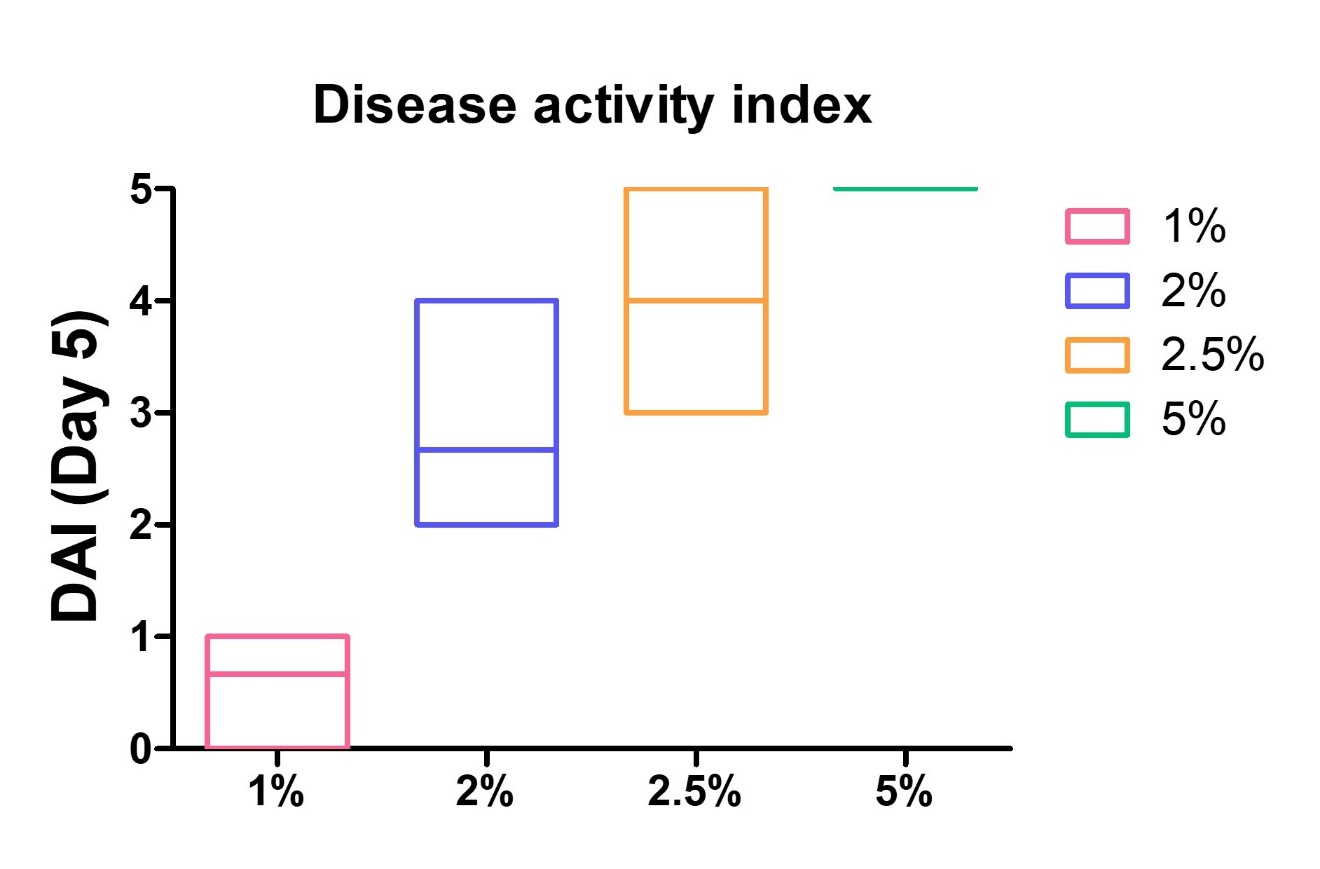

Supplement: Supplementary file 1 — Supplementary material [file mmc1.docx]
